# Brain activation in individuals suffering from bulimia nervosa and control subjects during sweet and sour taste stimuli

**DOI:** 10.3389/fpsyt.2023.1022537

**Published:** 2023-03-02

**Authors:** Daphna Bardin Armon, Atira Bick, Sharon Florentin, Sofia Laufer, Gabriel Barkai, Eytan Bachar, Talma Hendler, Omer Bonne, Shikma Keller

**Affiliations:** ^1^Psychiatry Department, Hadassah Medical Center, Faculty of Medicine, Hebrew University of Jerusalem, Jerusalem, Israel; ^2^Neurology Department, Hadassah Medical Center, Faculty of Medicine, Hebrew University of Jerusalem, Jerusalem, Israel; ^3^Psychiatry Department, Tel Aviv Sourasky Medical Center and Tel Aviv University Sackler Faculty of Medicine, Tel Aviv, Israel; ^4^Wohl Institute for Advanced Imaging, Tel Aviv Sourasky Medical Center and Tel Aviv University Sackler Faculty of Medicine, Tel Aviv, Israel

**Keywords:** bulimia nervosa, neuroimaging, taste, sweet, sour, eating disorders

## Abstract

**Introduction:**

Episodes of eating great quantities of extremely sweet and often aversive tasting food are a hallmark of bulimia nervosa. This unique eating pattern led researchers to seek and find differences in taste perception between patients and healthy control subjects. However, it is currently not known if these originate from central or peripheral impairment in the taste perception system. In this cross sectional study, we compare brain response to sweet and sour stimuli in 5 bulimic and 8 healthy women using functional magnetic resonance imaging (fMRI).

**Materials and methods:**

Sweet, sour and neutral (colorless and odorless) taste solutions were presented to subjects while undergoing fMRI scanning. Data were analyzed using a block design paradigm.

**Results:**

Between-group differences in brain activation in response to both sweet and sour tastes were found in 11 brain regions, including operculum, anterior cingulate cortex, midbrain, and cerebellum. These are all considered central to perception and processing of taste.

**Conclusion:**

Our data propose that sweet and sour tastes may have reward or aversion eliciting attributes in patients suffering from bulimia nervosa not found in healthy subjects, suggesting that alteration in taste processing may be a core dysfunction in bulimia nervosa (BN).

## Introduction

Symptoms of bulimia nervosa (BN) include repeated events of ingesting enormous quantities of food (termed “binge”) followed by inappropriate compensatory behaviors to avoid weight gain. During binges there is a feeling of uncontrol regarding quantity or nature of food ingested (1).

Taste perception is complex since it combines appearance, familiarity, odor, texture, and temperature of food and, for human beings, also the social, emotional and cognitive contexts under which it is eaten. Therefore, taste is considered a multimodal sense. Researchers to this date have not succeeded in revealing its anatomical pathways or functional circuitry (2).

Patients with BN exhibit preference for sweet taste (3–5), and do not show a decrease in craving (6) or pleasantness (7) after repeated ingestion of sweet food constituents. The ingested foodstuffs may also be aversive, and include frozen food, food picked up from garbage, spoiled food, etc (4). These anomalous eating patterns led researchers to seek an aberration in peripheral taste perception and cerebral representation and processing of taste stimuli in patients with BN.

Only a few functional imaging studies examined brain response to taste stimuli in patients with active BN. Women with (largely sub-threshold) BN showed trends for less activation than healthy controls in the left middle frontal gyrus, right posterior and mid dorsal insula, right precentral gyrus and left thalamus in response to consumption of chocolate milkshake compared to a tasteless solution (8). Data obtained from the same cohort (9) suggest that negative affect may increase the reward value of food for individuals with BN. Functional imaging studies conducted on recovered BN patients show (10) that individuals recovered from a bulimic-type eating disorders (ED) had significantly lower activation than controls in the right anterior cingulate cortex (ACC). A more recent study from the same group (11) found that women recovered from BN had a significantly elevated hemodynamic response to the taste of sucrose in the right anterior insula. Khalsa et al. (12) suggested that adults remitted from BN may have elevated reward-related brain activation in response to taste after having eaten, and this may underlie the tendency to eat beyond satiety. Studies looking at brain response to visual food presentation in BN reported that patients, compared with healthy controls, displayed increased activation in the medial orbitofrontal cortex (OFC) and the anterior cingulate and decreased activation in the inferior parietal lobe and the left cerebellum in response to food perceived as aversive (13).

The purpose of this study was to compare brain response to sugar, a pleasant taste, to sour, an aversive taste, in contrast to water, a neutral taste, between patients with BN and matched healthy controls. We believe patients with BN are impaired in brain processing and assignment of reward value to sweet taste. We hypothesized we will see reduced activation in BN patients in secondary associative taste areas such as the OFC and ACC in response to exposure to unpredictable sweet stimuli. We presented participants in the study with an aversive (sour) taste in an attempt to determine whether impairment in brain activation in BN is limited to sweet taste or may generalize to other tastes as well.

## Materials and methods

### Participants

Five women suffering from bulimia nervosa (bulimia group, BG) and 8 matched healthy women (control group, CG) participated in the study. Inclusion criteria were age 18–40, Body Mass Index (BMI) within normal range. Diagnosis of BN according to DSM-IV criteria. All participants were outpatients, with no current or past substance abuse, no systemic or neurological illnesses, and no history of head trauma. No psychiatric diagnosis, other than bulimia nervosa in the experimental group was accepted. Subjects were not on any medication other than oral contraceptives and smoked up to 10 cigarettes a day. Study was approved by our institutional review board and all participants signed an informed consent form.

Subjects were assessed using the Structured Clinical Interview for DSM-IV (SCID) (14) Eating Disorder Inventory 2 (EDI2) (15) and the Yale – Brown Obsessive compulsive scale (Y-BOCS) (16).

Subjects were instructed to fast from 24:00 on the night prior to the experiment. They were given a standard breakfast at 8 AM, consisting of one 3% plain yoghourt, one red apple, and one cup of tea/coffee with one teaspoon of sugar. Blood sugar levels were measured using an Elite© instant glucose meter. Subjects with readings out of normal range were excluded. Experiments commenced at 9 AM.

### Stimuli

Taste stimuli consisted of three flavors: sweet (0.3 M sucrose, 10%), sour (0.05 M citric acid, 1%), and mineral water. Taste stimuli were given to subjects first outside the scanner: Nine cups (3 per taste), each containing 5cc of colorless and odorless solution, were presented to subjects. Each cup was rated for pleasantness (most repulsive to most enjoyable) and for intensity (weakest to strongest) on a 100 mm visual analog scale. Inside the scanner stimuli were delivered into the subject’s mouth *via* sterile tubes (tubes consisted of 3.6 meters of BioMetrix© infusion line). Stimuli were administered manually, 0.5 cc of solution drip onto the subject’s tongue. Between taste conditions a wash of mineral water was used to prevent flavor mixing and diluting. “Taste” blocks lasted 24 s in which a bolus of stimulus was administered every 3 s. (8 boluses). “Wash” block took 12 s in which 4 boluses were given. Each functional imaging session consisted of two runs, each 10 min long. Stimuli presentation was pseudorandomly ordered to ensure that all stimuli appeared in equal number over both sessions.

### Magnetic resonance imaging acquisition

Scans were performed on a whole-body 3 T MRI scanner, General Electrics Medical Systems G3, with resonant gradient echoplanar imaging system. Before the experimental run anatomical images were acquired through T1-weighted 3D spoiled gradient echo (SPGR) sequence, with high resolution. SPGR scanning protocol consisted of FOV of 240, with a matrix of 256×256, voxel size 1 mm × 1 mm ×1 mm. No evidence of structural abnormalities were found in any of the participants.

The functional T2^*^-weighted scans were obtained in an oblique plane, according to a line determined anatomically from under the frontal lobe all the way under the fourth ventricle, including the whole of the pons (being a primary taste area). Functional scanning parameters were: TR = 2 s, TE = 30 ms, FA = 90°, imaging matrix = 64 × 64, FOV = 20 cm. 33 slices were obtained with slice thickness 3 mm and no slice gap. A functional run consisted of 300 scans (10 min). At the end of each scan a short anatomical scan was performed in order to help with future alignment of functional and anatomical data.

### Data analysis

Data preprocessing and co-registration were performed using the Brain Voyager 2000, 4.96, software package, while statistical analysis was performed using Brain Voyager QX 1.8 (Brain Innovation, Maastricht, Netherlands). First 6 volumes were removed to allow signal stabilization. Head motion and slice scan time corrections and high-pass temporal filtering in the frequency domain were applied in order to remove drifts and to improve signal to noise ratio. For all subjects head motion was <1 mm. Co-registration of individual anatomical and functional data, and normalization with respect to one common reference data set (17) were performed for all subjects. Spatial smoothing of 4 mm was applied for group comparisons.

Analysis was performed in two steps: In the first, whole brain analysis was performed to find regions sensitive to taste. As the aim was to assemble all regions that may be of interest a lenient approach was used and we included all regions showing an effect of taste in any of the groups and in any of the contrasts (sweet > neutral; sour> neutral). In the next step we extracted the data from these regions to identify between group differences.

**Figure 1:**
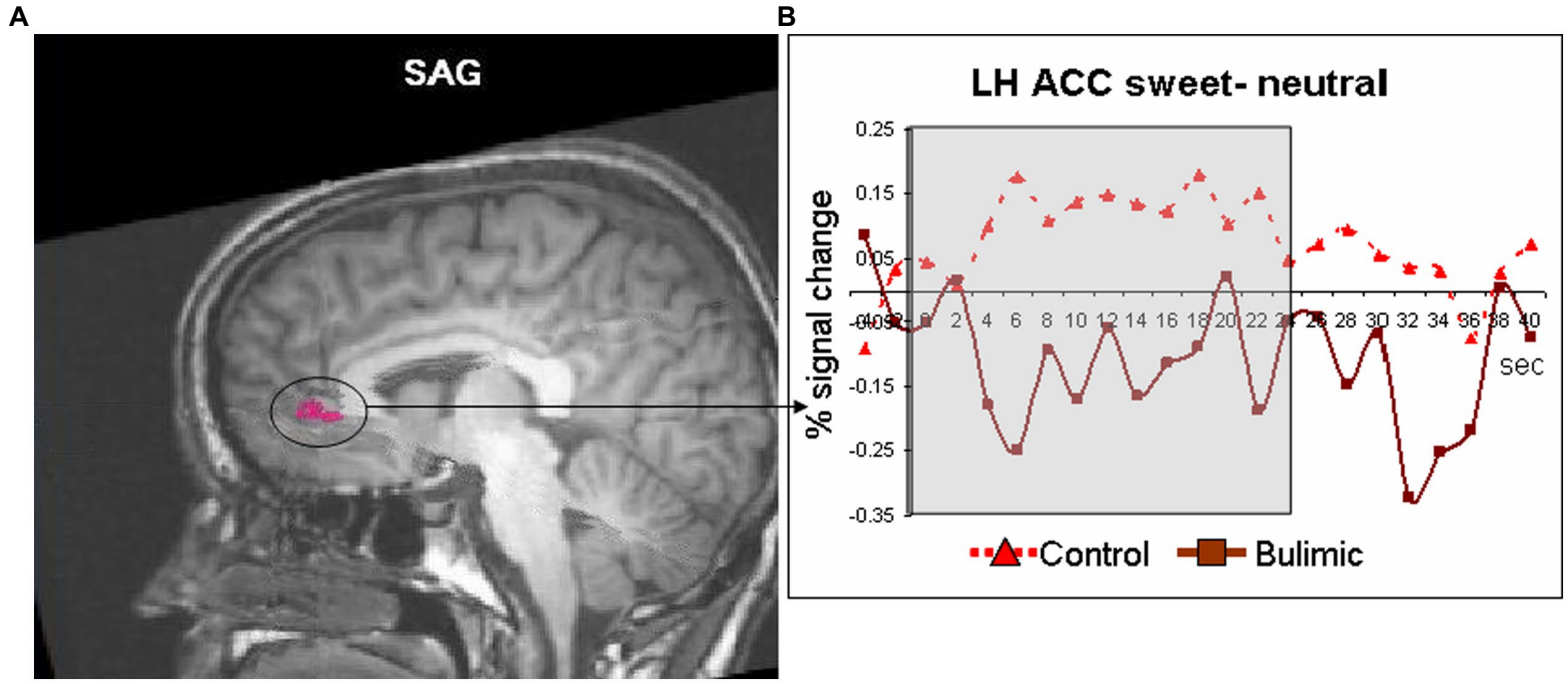
Significantly lower activation in the left anterior cingulate of bulimic subjects in response to sweet taste stimulation compared to controls. **(A)** Response to sweet compared to neutral taste in the anterior cingulate cortex (circled and marked in pink, displayed of T1 sagittal image) significantly differed between groups [*t*(11)=3.066, *p* < 0.01]. **(B)** Averaged time courses for each group.

A multi-study general linear model (GLM) was used to generate statistical parametric maps of both runs together. Group comparisons were calculated using random effects GLM. A minimum cluster size of 10 functional voxels was applied to all data. Thresholds used were corrected (*p* < 0.001) for cluster size. As regions involved in taste processing may be small, and this analysis is merely aimed in identifying regions involved in processing taste to be used in the following between group analysis a lenient cluster size was selected. For proof of concept, we first generated a statistical map of “taste” (both sweet>neutral and sour>neutral) for all subjects. Then, we used random effect GLM to detect brain regions significantly positively or negatively activated by either taste (sweet>neutral; sour>neutral) within each group (CG and BG). This was preferred over the between group whole brain analysis because we wanted to make sure that areas that are significantly different between groups are significantly activated in response to taste in at least one group. Regions found to be sensitive to taste in either group were used as regions of interest (ROIs) to evaluate the effect of bulimia on taste related regions. This allowed us to ensure that between groups effects were indeed in regions involved in processing taste. Activation from these regions was extracted for between group analysis using *t*-test. The averaged signal change during stimulus presentation was also calculated (Figure 1B).

## Results

Participants were 19–25 years old, female, with a BMI of 19–25, The two groups did not differ in age, height, weight, BMI and handedness, but significantly differed on most EDI (15) and Y-BOCS scales and sub-scales (16). The mean duration of BN was 6 years (SD = 4).

When solutions were presented outside the scanner (Table 1), a statistically non-significant trend (Mann–Whitney two group comparison) was seen in which the BG rated pleasure for sweet higher and for sour lower than the CG. BG seemed to enjoy sweet more and dislike sour more than CG. Both groups found the three different taste stimuli significantly distinct from one another, both in intensity and in pleasure ratings (a parametric Friedman test of ranks, *p* < 0.002 for CG, *p* < 0.015 for BG, data available on request).

**Table 1:** Mean values of intensity and pleasure for each flavor by group after tasting stimuli in cups outside the scanner, and Mann–Whitney two group comparison between groups.

	Sweet				Sour				Neutral			
	Intensity		Pleasure		Intensity		Pleasure		Intensity		Pleasure	
	BG	CG	BG	CG	BG	CG	BG	CG	BG	CG	BG	CG
Mean Value	51	52	74	60	78	77	8	22	21	9	57	50
Mann–Whitney U	18		10.5		16		10.5		13		13.5	
Significance	0.769		0.163		0.558		0.163		0.304		0.163	

No significant difference was found but a trend for higher pleasure for sweet taste and lower pleasure for sour in BG compered to CG was observed. Comparison of intensity and pleasure ratings given after tasting stimuli in cups. Mann Whitney U-two group comparison. BG, bulimic group; CG, control group.

Inside the scanner, taste (sweet and sour) vs. neutral analysis for all subjects revealed activation in the thalamus, midbrain and cerebellum in the right hemisphere (RH) and in the operculum, basal ganglia (BaGa), somatosensory cortex (SSC) and cerebellar regions in the left hemisphere (LH). Sour vs. neutral gave almost the same results as taste (sweet and sour) did. Sweet vs. neutral gave less areas of activation (data available on request). Group (BG/CG) and taste (sweet/sour) were then analyzed separately. All regions (except for the thalamus) found active in the “all subjects” analysis, were found active in at least one group/taste condition, as well as several additional regions (see Table 2). All were used as ROIs in a group by taste random effect analysis. Table 3 presents the 11 regions that significantly differed between the groups. In eight of these the BG had higher activation than the CG. In three regions activation was lower in the BG. Figure 1 shows significantly lower sweet vs. neutral response pattern in the left anterior cingulate of bulimic subjects compared to controls.

**Table 2:** Brain regions showing significant activation for taste (sweet/sour) vs. neutral, analyzed separately for each group.

	Sweet				Sour			
	RH		LH		RH		LH	
	BG	CG	BG	CG	BG	CG	BG	CG
Insula	33, 11, 8		−25, −7, 13	−31, 25, 3	34, 10, 7		−27, 21, −1	
Operculum*			−39, 35, 6**	−46, 17, 9				
ACC				**−10, 42, 8**				
SSC*					**47,** −52, 41**	57, −12, 30		−45, −17, 34
BaGa*	23, 10, 10	20, 7, 17	−24, 8, 9	−4, 2, 11	19, 4, 12	13, 3, 20	−18, −5, 15	
	10, 17, 7	11, 12, 12					−13, −24, −2	
Cuneus					**1,** −79, 3**		**−4,** −**69, 30**	
Midbrain*				**−9,** −**5,** −**9**	0, −12, 1			
MFG							**−45, 19, 34**	
IFG							**−52, 12, 23**	
IPG							**−49,** −**51, 32**	
POC		22–5,617						
Occipital				**−26, -76, 24**				
Cerebellum*		**19, -62, -30**				30, −55, −30	−25, −50, −25	−15, −45, −24
						9, −52, −31		

Bolded and underlined regions were significantly different between-groups in a random effect, region of interest analysis. Marked with * are Regions significantly activated in the taste vs. neutral comparison, all subjects analyzed together. Marked with ** are regions with higher activation in BG. Brain regions showing significant activation for taste (sweet/sour) vs. neutral, analyzed separately for each group. *p* < 0.005, corrected for cluster size. RH, right hemisphere; LH, left hemisphere; ACC, anterior cingulate cortex; SSC, somato sensory cortex; BaGa, basal ganglia; MFG, middle frontal gyrus; IFG, inferior frontal gyrus; IPG, inferior parietal gyrus; POC, posterior occipital cortex; Occipital, occipital cortex; BG, bulimia group; CG, control group. Bolded and underlined regions significantly different between-groups in a random effect, region of interest analysis.

* Regions significantly activated in the taste vs. neutral comparison, all subjects analyzed together.

** Higher activation in the BG.

**Table 3:** All brain regions showing preferences for taste in any of the groups.

	Whole brain within group analysis				ROI between group analysis	
Brain region	Group, flavor found in	Tal X,Y,Z	size	Average *p* value	Average statistic**	Average *p* value
LH Oper.	BG, sweet	39, 35, 6	979	0.003	−2.396	0.035
RH cuneus	BG, sour	1, −73, 3	548	0.003	−2.99	0.012
LH cuneus	BG, sour	−4, −69, 30	346	0.0025	−2.939	0.01
RH SSC	BG, sour	47, −52, 41	303	0.003	−3.22	0.008
LH MFG	BG, sour	−45, 19, 34	450	0.0035	−3.3	0.007
LH IFG	BG, sour	−52, 12, 23	339	0.003	−2.9	0.016
LH IPL	BG, sour	−49, 51, 32	402	0.003	−4.5	0.0009
RH cerebellum	CG, sweet	19, −62, −30	397	0.004	2.8	0.017
LH, ACC	CG, sweet	−10, 42, 8	288	0.0048	3.066	0.01
LH Occ’ c	CG, sweet	−26, −76, 24	487	0.004*	−3.2	0.008
LH midbrain	CG, sweet	−9, −5, −9	353	0.004	2.5	0.029

* Neutral > sweet.

** Negative numbers: BG > CG.

Table lists their location, size, the contrast they were found to be significant and the statistical value of the effect. For each region the results of the between groups analysis (statistical analysis and average *t*-value) are included. Regions found significant in ROI analysis between groups. RH, right hemisphere; LH, left hemisphere; Oper, operculum; SSC, somato sensory cortex; MFG, middle frontal gyrus; IFG, inferior frontal gyrus; IPG, inferior parietal gyrus; ACC, anterior cingulate cortex; Occ’ c, occipital cortex; Tal X,Y,Z, Talairach coordinate system; BG, bulimia group; CG, control group.

## Discussion

All regions activated by “taste” *per se* in this study were identified as such in previous studies (18, 19). Sour and sweet tastes activated both similar and different brain regions. This response pattern is not likely to be due to peripheral nervous system differences such as taste receptor damage following purging behavior, in which case the perceived differences would be the same for all flavors, but rather to impairment in central nervous system sensory and/or emotional processing circuitry.

Yeung et al. (20) performed connectivity meta-analysis of taste processing fMRI studies in healthy adults. Results revealed nine clusters activated by the effect of taste. Four involved the insula and the rest included the thalamus, pre and post central gyrus, hippocampus and caudate. Sweet taste contributed to all clusters while other tastes contributed to only some of the nine clusters (16). A systematic review of Chao et al. (5) found 15 studies that preformed fMRI to examine taste brain activation in ED. The vast majority of studies included only sweet stimuli. Due to differences in methodology and populations, strong conclusions could not be drawn. Neural responses differed when sweet taste stimuli were predictable compered to unpredictable stimuli. A general trend for reduced responsiveness during random application of taste but not during predictable applications was observed (5). In our study the application of taste was random. In eight regions the BG had higher activation than the CG. In three regions (such as the ACC, see Figure 1), activation was lower in the BG. In the same review, of nine taste preference studies, three compered BN to controls and found higher preference to sweet taste compered to controls (5), we found the same trend (non-significant) in our results.

Our finding of reduced activity in the ACC in the bulimic group replicates that of (10), who found reduced ACC activity in recovered BN in response to glucose, although reduced ACC activation in our study was found in the left hemisphere while (10) found a reduction on the right. Other imaging studies found altered serotonergic activity in cingulate regions in BN subjects (18). Moreover, (13) reported that presentation of pictures of food increased activity in the ACC and other regions in BN compared with CG subjects (13). Likewise, (19) report greater ACC activation in bulimic compared with healthy patients in response to visual presentation of high caloric food (19). This difference in ACC response between studies may reflect a difference in brain response to an actual perceptual sensation of taste versus response to a visual stimulus (and/or anticipation), or whether subjects were actively ill or recovered.

The ACC plays an important role in anticipation of reward (20). Regional cerebral blood flow in the ACC wsa reported to be inversely proportional to the desirability of chocolate (21), and cingulate activation was reported to be associated with cue-induced cocaine craving (22). Addiction-like cue reactivity has been described in bulimia nervosa (23). Thus, altered ACC activity may reflect a disturbance of taste reward expectancy in individuals with BN.

Brain activation in the inferior parietal region was higher in the BG relative to CG in response to sour taste and in the occipital cortex in response to sweet taste. (13, 15) found that patients with ED (Anorexia Nervosa and BN) showed decreased activation in inferior parietal lobe (IPL) and increased (13) or decreased (15) occipital activation after exposure to visual food stimuli relative to healthy control subjects. Activation of the IPL has previously been associated with appetitive and food-related behavior and satiation (24). Part of the IPL contains secondary and tertiary somatosensory areas (25) and the IPL is closely interconnected with the insula, the primary taste cortex, and receives both somatosensory and gustatory projections (26). Our findings indicate that posterior brain regions may be involved in the pathophysiology of eating disorders.

We found a region within the left operculum to be more active in the BG in reaction to sweet stimulus. In contrast, another region within the operculum and the insula was similarly activated in both groups. The operculum and insula are accepted as primary taste regions (26). Small et al. (14) found that the insula and operculum responded to pleasantness but not to intensity (14, 27) found opercular activation to be in correlation with pleasantness ratings of sweet stimuli (28). This is consistent with our finding showing higher activation in the left operculum in the BN group, as previous studies found that BN subjects prefer sweeter stimuli compared with CG (4). In these studies, intensity of taste did not differ between the groups, which could explain the lack of OFC and amygdala differences between the groups (14). An increased attribution of pleasantness to sweet stimuli, in combination with decreased ability to regulate affective behavior may be the beginning of a mechanism explaining binge eating, represented in, or stemming from brain alterations.

Cerebellar activation was elicited by both sour and sweet tastes. A significantly higher activation in a cerebellar sub-region was observed in the CG in response to the sweet stimulus. Not much has been reported on cerebellar activity in taste. Activity in the cerebellum has been found when showing pictures of food and pictures of emotional stimuli to bulimic subjects (13). The cerebellum has also been implicated in sending satiety signals (27). Small et al. (14) found the cerebellum responsive to intensity, irrespective of valence of taste stimuli. These last two findings offer an explanation for why in controls the cerebellum reacts more strongly to sweet taste than in bulimics, enabling bingeing of high intensity sweet foods which would have otherwise been aversive.

The striatum has been shown to receive inputs from the insula (29) and is hypothesized to mediate behaviors involving eating, particularly of highly palatable, high energy foods (30). Almost all imaging taste studies report findings in striatum and sometimes other regions of the basal ganglia, but most chose not to comment on these. We found activation in these regions in most subjects but did not find significant differences between groups.

## Strengths and limitations

This study is one of few studies comparing taste perception between subjects with active BN and healthy controls, and their brain response to sweet and sour stimuli. The small sample size (especially of the BG) is the major limitation of our study. Patients with BN were reluctant to undergo brain imaging of all sorts or take part in our taste evaluation paradigm.

Oral health complications associated with self-induced purging include symptoms such as hyposalivation, xerostomia, burning mouth syndrome and dysgeusia. These can all affect taste perception (31–33). Since oral status evaluation of BN subjects wasn’t performed in our study, this potential influence on taste perception wasn’t included in our analysis, and should be considered a limitation of our study.

A further limitation in our study may be that the neurological changes reported in our manuscript may be, at least in part, reflective of an underlying subclinical depressive condition (34). The oral burning sensation often reported by ED patients, secondary to oral mucosa atrophy, induced by deleterious nutritional choices and repeated self-vomiting episodes, could contribute to the evolution of depressed mood in this patient population (31).

Defining the ROIs separately in each group, while ensuring that all relevant regions will be included in analysis, may induce a bias towards “between group differences”. Indeed, in regions identified in the BG, activation was higher than controls, while in regions identified in CG, a mixed pattern was found. We recognize this is a limitation of the analysis we chose.

Swallowing during fMRI was shown to influence brain activity (35). While it was suggested to control for swallowing, this is not a standard practice and was not done in this study. As subjects were instructed to swallow at need we assume this was distributed randomly between conditions and therefore considered random noise. However swallowing may be influenced by taste (36) and may be influenced by BN as well. Further research is necessary to entangle the response to swallowing and taste and their interaction with Eating Disorders.

## Conclusion

Our findings imply that people with BN have aberrant sensitivity to the reward or aversion-inducing properties of sweet and sour tastes compared to healthy individuals. These appear to be associated with differences in brain activation, suggesting that impaired taste processing may represent a fundamental pathophysiology of BN. Further research with a larger number of subjects is needed to establish or refute these findings and reveal more subtle processes.

## Data availability statement

The raw data supporting the conclusions of this article will be made available by the authors, without undue reservation.

## Ethics statement

The studies involving human participants were reviewed and approved by Haddasah Hebrew University Medical Center Ethics Committee. The patients/participants provided their written informed consent to participate in this study.

## Author contributions

GB and OB performed the SCID to participants. DA, AB, and TH performed the experiments and contributed to data acquisition. EB contributed to the experimental design. OB, SK, and SF wrote the manuscript. SL and AB contributed to the interpretation of the results. All authors contributed to manuscript revision, read, and approved the submitted version.

## Conflict of interest

The authors declare that the research was conducted in the absence of any commercial or financial relationships that could be construed as a potential conflict of interest.

## Publisher’s note

All claims expressed in this article are solely those of the authors and do not necessarily represent those of their affiliated organizations, or those of the publisher, the editors and the reviewers. Any product that may be evaluated in this article, or claim that may be made by its manufacturer, is not guaranteed or endorsed by the publisher.
